# A Review of Fetal Development in Pregnancies with Maternal Type 2 Diabetes Mellitus (T2DM)-Associated Hypothalamic-Pituitary-Adrenal (HPA) Axis Dysregulation: Possible Links to Pregestational Prediabetes

**DOI:** 10.3390/biomedicines12061372

**Published:** 2024-06-20

**Authors:** Mathuli Ngema, Nombuso D. Xulu, Phikelelani S. Ngubane, Andile Khathi

**Affiliations:** School of Laboratory Medicine & Medical Sciences, University of KwaZulu-Natal, Private Bag X54001, Durban 4001, South Africa; 218022309@stu.ukzn.ac.za (M.N.); 215019278@stu.ukzn.ac.za (N.D.X.); ngubanep1@ukzn.ac.za (P.S.N.)

**Keywords:** type 2 diabetes mellitus, prediabetes, pregnancy, maternal HPA axis, fetal HPA axis, programming, fetal development, placenta, glucocorticoids, metabolic diseases

## Abstract

Research has identified fetal risk factors for adult diseases, forming the basis for the Developmental Origins of Health and Disease (DOHaD) hypothesis. DOHaD suggests that maternal insults during pregnancy cause structural and functional changes in fetal organs, increasing the risk of chronic diseases like type 2 diabetes mellitus (T2DM) in adulthood. It is proposed that altered maternal physiology, such as increased glucocorticoid (GC) levels associated with a dysregulated hypothalamic-pituitary-adrenal (HPA) axis in maternal stress and T2DM during pregnancy, exposes the fetus to excess GC. Prenatal glucocorticoid exposure reduces fetal growth and programs the fetal HPA axis, permanently altering its activity into adulthood. This programmed HPA axis is linked to increased risks of hypertension, cardiovascular diseases, and mental disorders in adulthood. With the global rise in T2DM, particularly among young adults of reproductive age, it is crucial to prevent its onset. T2DM is often preceded by a prediabetic state, a condition that does not show any symptoms, causing many to unknowingly progress to T2DM. Studying prediabetes is essential, as it is a reversible stage that may help prevent T2DM-related pregnancy complications. The existing literature focuses on HPA axis dysregulation in T2DM pregnancies and its link to fetal programming. However, the effects of prediabetes on HPA axis function, specifically glucocorticoid in pregnancy and fetal outcomes, are not well understood. This review consolidates research on T2DM during pregnancy, its impact on fetal programming via the HPA axis, and possible links with pregestational prediabetes.

## 1. Introduction

Fetal programming occurs during embryonic and fetal development, a vital stage during which tissues and organs are formed [1,2,3]. Many environmental cues, such as excess glucocorticoid exposure in utero, can contribute to various changes that include changes in molecular biological functions, such as receptor cell density or sensitivity, as well as alterations in metabolism or responses to physiological stressors [4,5]. Essentially, fetal programming refers to the process of sustaining or affecting a stimulus or impairment that occurs at a crucial point in its development [6,7]. Studies show that maternal diabetes, particularly type 2 diabetes mellitus (T2DM), with increased glucocorticoid (GC) levels, may be one of the common mechanisms through which glucocorticoid insults exert their programming effects [8,9]. Rapid economic development and urbanization, sedentary lifestyles, and the Westernized diet have led to a rising burden of 463 million (aged 20–79 years) adults living with T2DM in many parts of the world, especially in developing countries [10]. Although the weights of infants of diabetic mothers are generally skewed into the upper range, intrauterine growth restriction (IUGR), commonly diagnosed as low birth weight, occurs with concerning frequency in diabetic women, especially those with underlying hypertension, uncontrolled blood glucose levels, and vascular diseases [11,12]. T2DM has been shown to account for 30–50% of cases of pregestational diabetes during pregnancy [13].

Glucocorticoids (GC), such as cortisol in humans and corticosterone in rodents, are well-known for their role in glucose homeostasis in adult life [14]. The HPA axis regulates GC production through a feedback loop involving glucocorticoid receptors (GR) and mineralocorticoid receptors (MR) [14]. During pregnancy, this feedback mechanism ensures that GC levels are kept within a range that supports pregnancy while avoiding the adverse effects of hypercortisolism [15]. GC in pregnancy has also been shown to be essential in fetal maturation [5,16]. However, fetal GC load is usually regulated by 11β-hydroxysteroid dehydrogenase type-2 (11β-HSD2), a placental enzyme that inactivates GCs [17,18]. Increased maternal GC levels observed in T2DM and attenuating 11β-HSD2 expression potentially increase fetal exposure to GCs, slowing fetal growth and altering the gestational period [19,20,21]. Excessive glucocorticoid exposure in utero goes as far as altering the set-point and development of the offspring’s HPA axis that alternately reprograms the HPA axis, thus compromising its function after birth [22,23,24]. In addition, excess maternal or fetal corticosterone causes the downregulation of fetal GR and MR and impairs the feedback regulation of the HPA axis in both infancy and adulthood [25,26]. Cross-sectional research has also indicated a connection between lower birth weights, elevated cortisol levels, catch-up growth in the neonatal period, and adult obesity, which may be an indication of unfavorable adaptive responses until birth [27,28]. The association with low birth weight was first reported as a several-fold increase in the incidence of glucose intolerance and T2DM in adult men compared with those born with normal birth weight [29,30]. Furthermore, studies demonstrate that low birth weight has been associated with high risks of other non-communicable diseases (NCDs) such as hypertension, cardiovascular diseases, and mental disorders in adulthood, correlating with the concept of the Developmental Origins of Health and Disease (DOHaD) hypothesis [31,32]. The DOHaD, which emerged as a broadening of the “Barker hypothesis” and was named after epidemiologist David Barker, explains the scenario in which in utero maternal insults cause structural and functional alterations in fetal organs, extending postnatal life and increasing susceptibility to chronic disease in adulthood [33,34]. 

Prediabetes is characterized by impaired glucose metabolism, with glucose concentration above the optimal value but still below the diagnostic levels for T2DM [35]. In 2019, the prevalence of prediabetes was 373.9 million, with 15.3% undiagnosed, and it is expected to increase to 453.8 million by 2030 in parallel with increasing T2DM prevalence [36,37]. Studies show that prediabetes precedes T2DM, and it has been suggested that the onset of complications associated with T2DM begins during the prediabetic state, including myocardial injury, renal dysfunction, hormonal dysfunction, and dysregulation in the HPA axis function, among others [38,39,40,41]. The literature primarily reports alterations that occur in pre-existing T2DM pregnancies and fetal programming, while the changes in maternal pregestational prediabetes HPA axis function, specifically glucocorticoid and its influence on fetal outcomes, have not yet been explored. Therefore, this review consolidates research on T2DM during pregnancy, its impact on fetal programming via the HPA axis, and its possible links with pregestational prediabetes. The following section describes fetal programming, theories, and associated diseases. 

## 2. Fetal Programming

According to Barker (1995), the early life environment affects fetal growth and adds to disease susceptibility [42,43]. The developing baby adapts to an insult in utero, leading to long-term changes in form, physiology, and metabolism that are beneficial for survival [33,34]. Gluckman discovered that mismatches between early and later life circumstances might cause maladaptive alterations that raise the risk of a variety of cardiometabolic and psychiatric disorders as well as vulnerability factors, pertaining to the phenomena known as fetal programming [44,45]. Fetal programming occurs when the normal pattern of fetal development is disrupted by an abnormal stimulus or ‘insult’ applied at a critical point in in utero development [46,47,48]. According to the evidence for the Developmental Origins of Health and Disease (DOHaD) hypothesis, the antenatal period is a particularly vulnerable period of development in which exposure to adverse environments, such as glucocorticoid exposure, can have long-term or permanent effects on the offspring’s health trajectory [49,50]. Studies have shown that maternal HPA axis is crucial during fetal development [51,52]. However, maternal dysregulation in the HPA axis during pregnancy or before pregnancy has been shown to exert its programming effect, especially in the brain, notably in the HPA axis [53,54,55]. The following section details the physiological role of the maternal HPA axis in pregnancy, and its role in fetal development.

## 3. Role of the Maternal Hypothalamic–Pituitary–Adrenal (HPA) Axis in Pregnancy

**Figure 1:**
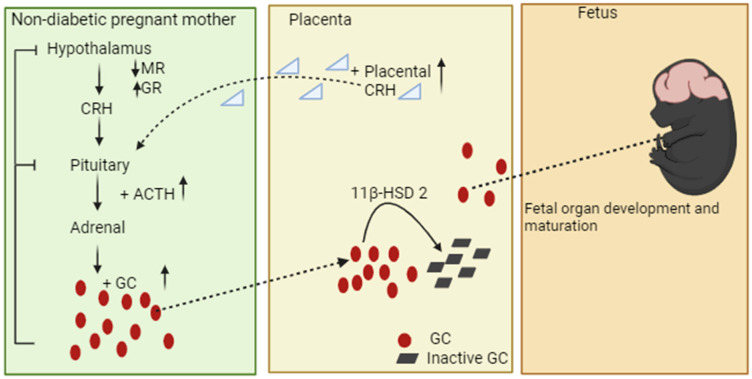
A schematic presentation of maternal HPA axis and GC signalling between mother, placenta, and fetus. Glucocorticoids tightly control HPA axis activity through glucocorticoid receptors (GR) and mineralocorticoid receptors (MR) in the pituitary and hypothalamus to inhibit CRH release, ACTH, and its own secretion [83,88]. In pregnancy, the placenta secretes large quantities of CRH into the maternal bloodstream as the pregnancy progresses, which promotes the production of GC [16,77]. Increased placental CRH secretion and GC also increase GR, promoting negative feedback and, therefore, maintaining the HPA axis activity in pregnancy. Nevertheless, the fetus is shielded from excess maternal GC exposure by the increased activity of 11β-hydroxysteroid dehydrogenase type 2 (11β-HSD2). The minimal transfer of GC from the placenta to the fetal compartment plays a vital role in the development of fetal organs, particularly the brain’s HPA axis and the maturation of the lungs.

The hypothalamic–pituitary–adrenal axis is a complex system of neuroendocrine pathways and feedback loops that functionally maintain physiological homeostasis through a synthesis of glucocorticoids (GCs). Active GCs are known as cortisol in humans and corticosterone in rodents [56]. The maternal HPA axis adapts during pregnancy, and regulates stress-related deleterious effects on the mother and offspring [57,58]. A non-diabetic pregnancy is a state of hyperactivity in the HPA axis and is also a state of hypercortisolism, especially towards late gestation [59,60,61]. The increased cortisol in late gestation is regulated by the placenta, an important source secreting the corticotropin-releasing hormone (CRH), which further enters the maternal pituitary gland via the hypophyseal portal circulation and enhances adrenocorticotropin (ACTH) synthesis and secretion into the peripheral circulation (Figure 1) [62,63]. ACTH increases glucocorticoid synthesis and secretion through the adrenal cortex in the kidney into the bloodstream in the course of pregnancy [64,65]. GC levels influence the hypothalamic CRH in a negative feedback loop, while the placental CRH is strongly stimulated by GC in a mechanism of a positive feedback loop [66,67]. In addition, studies show that high GC in pregnancy also plays a primary role in regulating fuel homeostasis. After the uptake of free cortisol from the circulation, cortisol increases the availability of potential fuel substrates by the mobilization of glucose, free fatty acids, and amino acids through the enhancement of hepatic gluconeogenesis and glycogenolysis [68,69]. Hence, research shows that GC contributes to insulin resistance, which is necessary to ensure that an adequate amount of glucose reaches the fetus for its growth and development [70]. 

Research shows that there are two types of corticosteroid receptors in the brain, namely glucocorticoid (GR) and mineralocorticoid (MR) receptors involved in the feedback regulation of the HPA axis [15,71]. The proper functioning of these receptors ensures that glucocorticoid levels remain within a range that supports pregnancy without causing undue stress to the mother or the fetus [72]. In the brain, MR binds to endogenous glucocorticoid with a higher affinity than GR, and, at basal concentrations of cortisol and corticosterone, MR is occupied while the GR remains largely unoccupied [73,74]. During times of elevated plasma glucocorticoid levels, such as during stress, increased occupation of GR helps to reduce the release of CRH and ACTH, ultimately lowering glucocorticoid production, and thereby regulating the function of the HPA axis. [75,76]. In pregnancy, MR mRNA expression in the hippocampus is unaltered, and GR gene expression is only modestly increased, which promotes negative feedback, maintaining HPA axis activity (Figure 1) and, hence, the diurnal secretion of cortisol is maintained throughout pregnancy [16,77]. Furthermore, studies show that late pregnancy (the last week, in the rat) is associated with a substantial reduction in HPA axis responses to both psychological and physical stressors in several species [77,78,79]. This adaptation is considered to buffer the impact of stress by reducing fetal exposure to maternal glucocorticoid, thus minimizing the risk of detrimental glucocorticoid programming [16,80]. 

Furthermore, studies show that glucocorticoids are lipophilic and can readily pass through the placental barrier by simple diffusion [81,82]. While glucocorticoid’s most well-known function is to stimulate differentiation and functional development of the lungs, glucocorticoids also play crucial roles in the development of several other organ systems, including the HPA axis [51,52]. However, 11β-hydroxysteroid dehydrogenase type 2 (11β-HSD2) acts as a barrier enzyme to control the passage of glucocorticoids from the mother to the fetus and protects the fetus from the much higher maternal levels of glucocorticoids [83,84]. This enzyme is found on both placental sides of the syncytiotrophoblast [84,85]. It metabolizes active glucocorticoids (cortisol in humans, and corticosterone in rats) into inactive glucocorticoids, thereby shielding the fetus against excessive glucocorticoid exposure from the mother, as shown in (Figure 1) [85,86]. Although the placenta metabolizes a significant proportion of cortisol (80–90% during gestation), excess cortisol may reach the fetus, and the ‘barrier’ can be further weakened by maternal high maternal glucocorticoid or placental dysfunction, which is commonly caused by increased oxidative stress, resulting in hypoxia, allowing for the increased transfer of glucocorticoids from the mother to the fetus [16,54,87]. The following section describes T2DM, prevalences, pregestational consequences associated with fetal programming, and associated diseases in adulthood.

## 4. Changes in the HPA Axis in T2DM Pregnancies Associated with Fetal Programming

T2DM accounts for 90–95% of all diagnosed diabetes mellitus (DM) cases and is regarded as a complicated and multifaceted illness caused by a mix of genetic and environmental risk factors [89]. T2DM is characterized by insulin resistance and inadequate β-cell responsiveness to glucose stimulation [90]. Globally, the International Diabetes Federation (IDF) estimated that by 2045, 629 million are expected to have T2DM aged 20–70 years [91]. Swift urbanization, marked by the uptake of unhealthy, calorie-rich diets and sedentary lifestyles, has played a role in the progressively rising prevalence of T2DM, particularly among females compared to males, and the prevalence rises with age [92,93]. Research suggests that, while T2DM is often associated with macro-and microvascular complications, individuals with poor management of everyday stress is also associated, in diabetic patients, with the constant activation and disrupted regulation of the HPA axis, showing a similar resemblance to maternal stress, accompanied by high levels of glucocorticoids [94]. Champaneri et al. similarly found high cortisol (hypercortisolism) levels throughout the day in diabetic women [95]. Established diabetes mellitus, either type 1 or 2, is the most common pre-existing medical condition in pregnant women at younger ages, resulting in an increasing proportion of pregnancies complicated by diabetes [96,97,98]. In some areas, pregnant women with T2DM now outnumber those with type 1 diabetes (T1DM) [99]. Research indicates that pregnant women with T2DM exhibit comparable patterns to those observed in maternal obesity and in depressed and stressed pregnant women [100,101]. These patterns involve the prolonged activation and dysregulated function of the HPA axis with elevated glucocorticoid levels [100,101].

The pathophysiology of fetal growth in the context of T2DM pregnancy is intricate and multifaceted [102,103]. However, the complications of diabetes affecting the mother and fetus are well-known [104,105,106]. Maternal complications include preterm labour, nephropathy, vascular diseases, caesarean section, postoperative wound complications, uncontrolled hyperglycemia, and increased oxidative stress, among others [107]. Fetal complications include fetal wastage from early pregnancy loss or congenital anomalies, macrosomia, shoulder dystocia, stillbirth, and intrauterine growth restriction (IUGR), among others [108,109]. 

Approximately 20% of pregnant women with diabetes experience gestational hypertension and/or preeclampsia [110]. The individuals most susceptible to these conditions are those who have pre-existing microvascular complications such as microangiopathy, hypertension, or inadequate control of blood glucose levels, which also contribute to endothelial dysfunction [110,111]. These complications have been shown to induce a reduction in trophoblast proliferation, delaying placental growth and development, particularly in the first few weeks of gestation [112]. This mechanism suggests the presence of dysregulation of trophoblast invasion by the diabetic environment, leading to decreased placental perfusion, which results in placental dysfunction [113]. 

**Figure 2:**
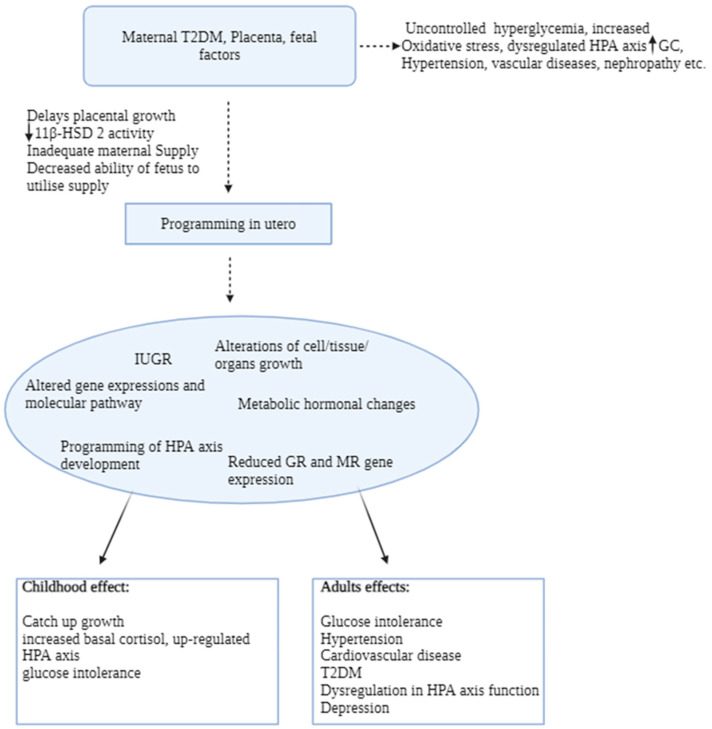
The schematic diagram presents the summary of maternal T2DM pregnancy complications, leading to fetal consequences in utero that persist until adulthood.

Studies show that placental dysfunction is associated with relatively low placental 11β-HSD2 activity, therefore increasing active maternal GC to the fetus’s bloodstream [114,115]. Overexposure to glucocorticoids during fetal development causes modifications in the expression of various cytostructural proteins, receptors, enzymes, ion channels, and growth factors [116]. These modifications result in changes in tissue structure, biochemical composition, metabolism, and hormone responsiveness, impacting the functionality of several fetal organ systems [117,118]. A study has discovered a correlation between maternal diabetes accompanied by elevated cortisol levels and alterations in the development of the brain’s cortical neuroendocrine system by reducing the number of hippocampal neurons [119]. Nevertheless, the precise molecular and cellular process via which diabetes during pregnancy impacts brain development remains unknown [120]. Consequently, glucocorticoids trigger physiological processes that have little or insignificant roles in utero but which become crucial at birth, such as the HPA axis [121]. The HPA axis and its key limbic regulator, the hippocampus, are particularly sensitive to glucocorticoids and their perinatal programming actions [52,122]. Previous studies show that glucocorticoid excess exposure during fetal development programs has specific effects on the brain, notably the HPA axis [123,124]. This exposure changes its development, sensitivity, and activity in utero, relatively stressing its growth as the HPA axis begins to develop during the embryonic stage and continues to mature throughout pregnancy [25,125]. As a result, studies show that prenatal glucocorticoid exposure permanently increases basal plasma corticosterone levels in adult rats [122,126]. This was because the density of both types of corticosteroid receptors, GRs and MRs, are permanently reduced in the hippocampus, changes which are anticipated to attenuate HPA axis feedback sensitivity from maternal stress shown in Figure 2 [122,126,127].

In addition, studies show that fetal exposure to excess maternal GC relative to early increases in the fetal GC concentration also triggers tissue differentiation and reduces accretion in the fetus [5]. As a result, the overall rate of maturation and growth declines as GC concentrations rise in the fetus toward term and in response to adverse intrauterine conditions, resulting in growth-retarded fetuses recognized as having IUGR [12,128]. The term intrauterine growth restriction (IUGR) refers to neonates whose birth weight and length fall below the tenth percentile for their gestational age [129,130]. IUGR is a common antenatal diagnosis; nevertheless, some of these fetuses, particularly those who were not checked during pregnancy, may be discovered only after birth [129,131]. The primary diagnostic criteria for IUGR include low birth weights (LWs), a surrogate marker of an adverse intrauterine environment, and subsequent cardio-metabolic disease and mental health problems [132,133]. 

On the other hand, the brain is heavily reliant on glucose for energy, and mammals have redundant systems for controlling glucose production [134]. As a result, it is possible that altered hypothalamus function may promote the dysregulation of peripheral glucose metabolism, leading to insulin resistance or T2DM in adulthood [135]. Research conducted by Hales et al. revealed a several-fold higher incidence of glucose intolerance and T2DM in adult men who were born with low birth weight as opposed to those who were born with normal birth weight, which established the first link between low birth weight and the development of T2DM [29]. A study in rats showed that the smallest fetuses with the largest placentas had lower placental 11β-HSD2 activity and were projected to have the highest adult blood pressures [17,136]. Heightened HPA axis activity, particularly with increased ACTH and high plasma glucocorticoid levels, is seen in children and adults who were born underweight [137,138]. In addition, previous research has indicated that infants born with lower birth weights undergo catch-up growth within the initial two years of life [139,140]. This process is seen as a means to offset their genetically predetermined growth patterns [140]. Catch-up growth is also observed in other aspects of growth, such as changes in body weight and body composition [141]. As per the theory of DOHaD, the rapid catch-up growth experienced by low-birth-weight infants in their early years is associated with various metabolic conditions such as obesity, hypertension, cardiovascular diseases, metabolic syndrome, and endothelial dysfunction later in adulthood shown in Figure 2 above [142,143]. Men were found to be more likely to develop cardiovascular disorders than women born with IUGR due to hormonal differences, as men had lower levels of estrogen, which has protective effects on the circulatory system and may contribute to women’s decreased risk of cardiovascular disease [144,145]. A prior study discovered that a combination of placental weight and birth weight predicts the risk of high blood pressure and hypertension in men and women around the age of 50 [137,146]. People who were babies with large placentas had the highest blood pressure and a higher risk of hypertension [147]. Both the Barker and DOHaD hypotheses support these theories. 

## 5. Prediabetes

Prediabetes is a condition in which blood glucose levels are abnormally high, but do not meet the diagnostic criteria for type 2 diabetes mellitus (T2DM) [148]. Prediabetes can be identified by at least two of these characteristics: impaired fasting glucose (IFG), impaired glucose tolerance (IGT), or high glycated hemoglobin A1c (HbA1c) [149]. IFG patients have significant hepatic insulin resistance with normal skeletal muscle values and poor glucose suppression, which causes hyperglycemia during fasting due to impaired insulin secretion or reduced sensitivity of β-cells to glucose stimulation [150]. IGT mainly impacts muscle insulin resistance, with minimal effects on liver insulin sensitivity. The reduced glucose absorption observed in individuals with impaired glucose tolerance (IGT) contributes to postprandial hyperglycemia [151,152]. This is primarily due to pancreatic β-cell dysfunction, resulting in inadequate secretion of insulin to counteract elevated glucose levels and stimulate a response in insulin-targeted peripheral tissues [153,154]. Lastly, IFG Individuals have a poor early insulin response during the oral glucose tolerance test (OGTT), but improve insulin secretion during the second phase, which is one of the reasons why prediabetes is frequently undetectable [155]. As a result, the American Diabetes Association (ADA) recommendations were changed in 2003 to identify patients with prediabetes based on the following values. Fasting plasma glucose levels range from 5.6 mmol/L to 6.9 mmol/L, whereas IGT values recorded after OGTT range from 7.8 mmol/L to 11.0 mmol/L [156,157]. Glycated haemoglobin (HbA1c) levels between 5.7% and 6.4% are used as an additional diagnostic criterion for prediabetes [158,159]. Prior to the diagnosis of pre-diabetes, there is a presence of insulin resistance and malfunctioning of pancreatic β-cells [153,154]. Studies show that a diet high in saturated fats, high in carbohydrates, or high in fructose contributes to the development of intermediate hyperglycemia [160,161]. In addition, studies also show that these caloric foods lead to elevated triglycerides and increased release of free fatty acids (FFA) from adipocytes into circulation, which is accompanied by decreased FFA uptake by adipocytes in insulin-dependent tissues, promoting insulin resistance and dyslipidemia [162,163]. These actions result in increased circulating FFA levels and FFA flux to the liver, which stimulates increased production and secretion of atherogenic very-low-density lipoprotein and small dense low-density lipoprotein particles, and reduced high-density lipoprotein cholesterol (HDL-C) levels, increasing the risk of microvascular and macrovascular diseases [162,164]. In addition, in the condition of insulin resistance, normal levels of insulin in the blood would be unable to elicit a reaction in the peripheral tissues that are targeted by insulin due to a decrease in the number of insulin receptors on the surface of cells, including muscle cells [165,166]. With fewer receptors available, the cells become less responsive to insulin, reducing their ability to take up glucose [90]. Consequently, the β-cells of the pancreas react by producing additional insulin to counterbalance the increased glucose levels [167]. When the β-cells fail to secrete sufficient insulin to counteract insulin resistance, the blood glucose levels commence fluctuating, leading to intermediate hyperglycemia and hyperinsulinemia, leading to further alterations in β-cell function [168]. 

### 5.1. Prevalence of Prediabetes

The prevalence of prediabetes has grown worldwide, and in 2019, the International Diabetes Federation estimated the worldwide prevalence of prediabetes to be 373.9 million, with 15.3% undiagnosed according to studies [36,37]. It is also projected that by 2030, approximately 453.8 million people will have prediabetes [37,169]. The prevalence of prediabetes is anticipated to increase to 8.3% of the global adult population, equivalent to an estimated 587 million individuals by the year 2045 [170]. Studies show that prediabetes is frequently undetected due to its often-asymptomatic nature in its early stages; hence, most humans tend to unknowingly bypass the prediabetes stage to overt T2DM [159,171]. In addition, studies show that the increase in prediabetes prevalence is due to rapid urbanization, increasingly sedentary lifestyles, and unhealthy eating habits [172,173]. As a result, a retrospective study in a rapidly urbanizing area such as Durban, South Africa, indicated that 68% of the individuals are prediabetic in the sample population between the ages of 20–45 years, with 51.0% of the study population being women [174]. This suggests that women of childbearing age are also affected by the global rise in prediabetes [175]. Furthermore, studies show that people with prediabetes have a two-fold increased likelihood of developing T2DM [176,177]. Moreover, studies show that complications associated with T2DM are already evident in some people with prediabetes; these complications include myocardial injury, renal dysfunction, hormonal dysfunction, and dysregulation in HPA axis function, among others [38,39,40,41].

### 5.2. HPA Axis Function in Prediabetes

Animal models have been observed to mirror human disease conditions, making them extensively utilizable for studying physiological systems and human disease states [178,179]. A high-fat, high carbohydrate and 15% fructose diet-induced animal model of prediabetes has been found to mimic the human condition [38]. In addition, this animal model showed dysregulation in the functioning of the HPA axis in the prediabetic state, as shown by elevated basal corticosterone and impaired regulation of their glucocorticoid receptor (GR) and mineralocorticoid receptor (MR) in male prediabetic rats [39]. At present, there have been no investigations to show whether this phenomenon also exists in female prediabetic rats. Furthermore, if present, this raised the question of whether maternal basal corticosterone and ACTH levels in prediabetic dams may impact fetal HPA axis development. 

## 6. Possible Links of Prediabetes with T2DM Pregnancies in Association with the HPA Axis

Both T2DM and prediabetes result from insulin resistance, leading to impaired glucose tolerance and chronic hyperglycemia [154,180]. Several studies have shown that during pregnancy, increased maternal serum glucocorticoids (GC), observed in T2DM and maternal stress, may cross the placenta, overwhelming the protective placental barrier [11,30]. Conversely, research shows that pre-existing metabolic disorders associated with T2DM, such as hypertension, renal disease, and maternal microangiopathy during pregnancy, decrease trophoblast proliferation [110,111]. This delays placental growth and its ability to supply the fetus with enough nutrients and oxygen, resulting in fetal hypoxia and inadequate nutrition supply [112]. Decreased placental function is linked to placental dysfunction and relatively low activity of placental 11β-HSD2 [115,118]. These complications have been associated with increasing the vulnerability of the fetus during this period to unwanted programming effects such as increased transfer of active maternal cortisol to the fetal compartments [115,118]. Early maternal GC exposure to the fetus has been associated with alterations to the balance of both GR and MR development in the fetal brain, leading to changes in gene expression patterns and neural circuit formation, evidenced by low GR and MR expression in offspring after birth, even in both basal and stressed animals [126]. Moreover, studies show that excessive GC exposure during critical periods of development could program the fetal HPA axis to be hyper-responsive, beginning in utero and persisting later in life, leading to outcomes such as the prolonged increases in ACTH and GC seen in adulthood, potentially predisposing the offspring to diseases such as depression, cardiometabolic disorders, or T2DM [53]. Furthermore, studies suggest that excessive maternal GC exposure or reduced placental function during pregnancy is associated with reduced fetal growth, contributing to intrauterine growth restriction (IUGR), commonly diagnosed at birth as low birth weight [12,128]. Low-birth-weight offspring have been associated with HPA axis hyperactivity, glucose intolerance, hypertension, obesity, and greater risks for developing depression, anxiety, T2DM, and cardiovascular diseases in adulthood, especially when there was catch-up growth in the first 2 years, as supported by the DOHaD hypotheses [49,50]. 

There are various possible causes that contribute to IUGR or an unfavourable hostile environment during pregnancy, including preeclampsia and hypertensive disorders that have also been associated with greater risk in T2DM pregnancies [110]. Prediabetes, which often precedes the onset of T2DM, has been shown by various studies to be the genesis of complications associated with T2DM, including myocardial injury, renal dysfunction, hormonal dysfunction, and dysregulation in HPA axis function, among others [38,39,40,41]. A recent study by Ludidi and colleagues showed that prediabetes is a risk factor for developing pre-eclampsia, similar to T2DM pregnancy [181]. In addition, since prediabetes often goes undiagnosed, this suggests that there is a population of people unaware of their elevated risk of developing hypertension and preeclampsia [181]. Therefore, the presence of prediabetes in pregnancy might increase the likelihood of IUGR and impaired glucose tolerance in offspring. With the increasing prevalence of prediabetes, especially in women of childbearing age, there is an increased possibility of pre-gestational, gestational, and fetal outcome consequences. However, there have been no studies that have looked at how maternal prediabetes affects the HPA axis along with fetal outcomes (summarised in Table 1 below). Conducting preliminary investigations, generating hypotheses, carrying out invasive procedures, collecting tissue samples, and understanding the fundamental mechanism of pregnancy-related disorders would be ethically or practically challenging in humans. As a result, most primary studies are recommended to begin with animal models; hence, this study recommends that future studies focus on pregestational prediabetes, detailing its effects on maternal HPA axis function and its influences on fetal outcomes in Sprague Dawley rats.

## 7. Conclusions

This review paper highlights the significant impact of the dysregulation of the maternal HPA axis during pregnancy, particularly elevated glucocorticoid levels, on fetal growth and programming, with potential implications for HPA axis development in the fetus seen in T2DM. It discusses the possible links between prediabetes and T2DM pregnancies relative to impaired HPA axis function. Women with type 2 diabetes represent high-risk groups during pregnancy. As the incidence of diagnosed diabetes and prediabetes continues to increase, especially at young ages, the number of women with diabetes or prediabetes in pregnancy may also continue to increase. However, further research is needed to understand the effects of pregestational prediabetes on the maternal HPA axis and its impact on fetal outcomes. This could further underscore the importance of a continued investigation into the complex interplay between maternal metabolic health, HPA axis regulation, and fetal development to inform clinical management and improve pregnancy outcomes. 

## Figures and Tables

**Table 1:** Summarizes possible links of prediabetes with T2DM pregnancies in association with the HPA axis.

Prediabetes	T2DM
Prediabetes is characterized by the concurrent occurrence of insulin resistance and β-cell dysfunction, which are abnormalities that occur prior to the detection of changes in glucose levels [150,182].	T2DM, a condition marked by deficient insulin secretion by pancreatic islet β-cells, tissue insulin resistance (IR), and an inadequate compensatory insulin secretory response [183].
Studies show that young adults from 25–45 are diagnosed with prediabetes. The number of individuals with prediabetes is expected to rise to around 587 million by 2045 [170,174].	It is projected that, by 2045, the number of people with type 2 diabetes mellitus (T2DM) will reach 629 million people aged 20–79 years, respectively [91].
This study shows that a prediabetic diet-induced animal model showed dysregulation in the functioning of the HPA axis, associated with elevated basal corticosterone and impaired regulation of the glucocorticoid receptor (GR) and mineralocorticoid receptor (MR) in male prediabetic rats animals [39].	Patients with diabetes present alterations of the HPA axis negative feedback, suggestive of the impairment of corticosteroid receptor sensitivity, which is associated with high levels of glucocorticoids and ACTH [94].
Pre-eclampsia has been demonstrated to be associated with an increased risk in those with prediabetes [181]. Studies show that prediabetes is also linked to the early development of complications shown in T2DM. Therefore, the occurrence of prediabetes during pregnancy may increase the likelihood of exacerbated maternal dysregulation in HPA axis function, leading to fetal adverse consequences.	Research indicates that T2DM pregnancy with uncontrolled hyperglycemia, hypertension, elevated glucocorticoids, and an increased risk of pre-eclampsia is associated with higher rates of complications for both the mother and the baby [110,111]. The complications include IUGR, altered gene expression, HPA axis programming, glucose intolerance, increased risk of developing T2DM, and cardiovascular diseases, among others [67,137,184,185,186].

Nevertheless, no research has been conducted to investigate the impact of maternal prediabetes on the HPA axis in relation to fetal outcomes in pregnancy.
